# Colon impairments and inflammation driven by an altered gut microbiota leads to social behavior deficits rescued by hyaluronic acid and celecoxib

**DOI:** 10.1186/s12916-024-03323-0

**Published:** 2024-04-29

**Authors:** Oryan Agranyoni, Debpali Sur, Sivan Amidror, Nuphar Shidlovsky, Anastasia Bagaev, Nissan Yissachar, Albert Pinhasov, Shiri Navon-Venezia

**Affiliations:** 1 Department of Molecular Biology and the Dr. Miriam and Sheldon G. School of Medicine, Ariel University, Ariel, Israel; 2 The Goodman Faculty of Life Sciences, Bar-Ilan Institute of Nanotechnology and Advanced Materials, Bar Ilan University, Ramat Gan, Israel

**Keywords:** Social behavior, Gut microbiota, Colon mucin, Tregs, SCFAs, Gut permeability, Colon inflammation, Depressive-like behavior, Hyaluronic acid, Celecoxib

## Abstract

**Background:**

The exact mechanisms linking the gut microbiota and social behavior are still under investigation. We aimed to explore the role of the gut microbiota in shaping social behavior deficits using selectively bred mice possessing dominant (Dom) or submissive (Sub) behavior features. Sub mice exhibit asocial, depressive- and anxiety-like behaviors, as well as systemic inflammation, all of which are shaped by their impaired gut microbiota composition.

**Methods:**

An age-dependent comparative analysis of the gut microbiota composition of Dom and Sub mice was performed using 16S rRNA sequencing, from early infancy to adulthood. Dom and Sub gastrointestinal (GI) tract anatomy, function, and immune profiling analyses were performed using histology, RT-PCR, flow cytometry, cytokine array, and dextran-FITC permeability assays. Short chain fatty acids (SCFA) levels in the colons of Dom and Sub mice were quantified using targeted metabolomics. To support our findings, adult Sub mice were orally treated with hyaluronic acid (HA) (30 mg/kg) or with the non-steroidal anti-inflammatory agent celecoxib (16 mg/kg).

**Results:**

We demonstrate that from early infancy the Sub mouse gut microbiota lacks essential bacteria for immune maturation, including *Lactobacillus* and *Bifidobacterium* genera. Furthermore, from birth, Sub mice possess a thicker colon mucin layer, and from early adulthood, they exhibit shorter colonic length, altered colon integrity with increased gut permeability, reduced SCFA levels and decreased regulatory T-cells, compared to Dom mice. Therapeutic intervention in adult Sub mice treated with HA, celecoxib, or both agents, rescued Sub mice phenotypes. HA treatment reduced Sub mouse gut permeability, increased colon length, and improved mouse social behavior deficits. Treatment with celecoxib increased sociability, reduced depressive- and anxiety-like behaviors, and increased colon length, and a combined treatment resulted in similar effects as celecoxib administered as a single agent.

**Conclusions:**

Overall, our data suggest that treating colon inflammation and decreasing gut permeability can restore gut physiology and prevent social deficits later in life. These findings provide critical insights into the importance of early life gut microbiota in shaping gut immunity, functionality, and social behavior, and may be beneficial for the development of future therapeutic strategies.

**Supplementary Information:**

The online version contains supplementary material available at 10.1186/s12916-024-03323-0.

## Background

Social interactions are critical for the survival and development of animals and humans [1], playing a fundamental role in everyday life, and greatly influencing well-being and quality of life [2]. The impact of social interactions and their deficits are relevant for the development and manifestation of a wide spectrum of disorders, including anxiety and depression [3–5]. Accumulating evidence demonstrates the role of genetic and environmental factors in the development and establishment of social behavior [6].

Numerous human and animal studies have demonstrated that pathways underlying inflammatory and stress responses may play an essential role in the etiopathology of social deficits, depression, and anxiety [7–9]. Inflammation can directly or indirectly affect mental health, thus substantially increasing asocial behavior [8, 10]. For example, humans who received an endotoxin derived from *Escherichia coli* developed an immune reaction that resulted in emotions of depression and social disconnection [9, 11]. On the other hand, social defeat has been shown to lead to monocyte-mediated exacerbation of gut inflammation [12].

Moreover, social interactions have been correlated with the gut microbiota composition [13]. Fecal microbiota transplantation (FMT) from specific-pathogen-free to germ-free (GF) mice increased GF mice social behavior [14]. Social defeat, which leads to anxiety- and depressive-like behaviors, has been associated with changes in the alpha-diversity of the mouse gut microbiota and reduced the relative abundance of various bacterial genera [15]. Similarly, rat offspring from antibiotic-treated mothers demonstrated decreased social performances [16].

Furthermore, the gut microbiota plays a crucial role in the development and functionality of the immune system [17]. Studies in GF animals revealed that the lack of gut microbiota caused a significant immune system deficiency, including reduction of CD4+ T cells, specifically T regulatory cells (Tregs) [18]. Intestinal bacteria, such as *Lactobacillus*, *Bifidobacterium*, *Bacteroides*, and *Clostridium* [19], and their metabolites, butyric, and propionic acids, have been shown to activate Treg cells in various mouse or cell culture models [20]. Moreover, mouse offspring from high-fat-diet-fed mothers exhibited decreased social interaction which was improved with *Lactobacillus rhamnosus* probiotic treatment [21]. Although the gut microbiota may shape brain function by modulating the immune system [22], the comprehensive roles of the gut microbiota in predisposing the development of behavior deficits are still scarce.

To study the molecular and behavioral components of social behavior, we developed a mouse model with strong features of dominance (Dom) and submissiveness (Sub), essential elements of social behavior [23]. These Dom and Sub mice were derived from a Sabra mouse lineage using a selective breeding approach based on a social interaction food competition, the dominant-submissive relationship (DSR) test [23]. Using behavioral and pharmacological approaches, we previously demonstrated that Dom and Sub mice possess different cognitive and learning capabilities [24]. Moreover, compared to Dom mice, Sub mice exhibit strong stress sensitivity [25], along with depressive-like and anti-social characteristics [26], and systematic inflammation, demonstrated by higher- IL-6 and IL-1b serum levels [27].

Recently, we reported that adult Sub mice also exhibit an altered gut microbiota composition compared to Dom and Sabra mice [28], which was sex-independent. The involvement of the gut microbiota in the phenotype of Dom and Sub mice was proven by an FMT into GF mice, which demonstrated that Sub-transplanted mice acquired depressive-like and anti-social behaviors, alongside with increased inflammatory profile, typical for the Sub donor features [28]. The gut microbiota may have even been a unit of selection in Sub and Dom selective breeding scheme. The multi-system differences between Dom and Sub mice led us to hypothesize that alterations in the Sub mouse gut microbiota in early infancy, influence the developing gut immunity, leading to increased gut permeability and systemic inflammation, all of which may play a role in their social behavior deficits.

Here, we provide strong evidence that the gut microbiota mediates colonic and systemic inflammation from early infancy and is critical for the establishment of the altered behavioral and metabolic profiles of Sub mice. We studied the inflammatory and functional colonic alterations in Sub mice using age-dependent gut microbiota and colon physiology analyses, together with comprehensive immune cell and short chain fatty acid (SCFA) analyses in adulthood. We demonstrate that, alongside their altered gut microbiota, Sub mice possess higher gut permeability together with abnormal colon physiology, hyper-mucin secretion, and lower Tregs and bacterial SCFA levels. Furthermore, treating Sub mice with a gut permeability modulator agent or an anti-inflammatory drug therapy [29] rescued Sub mouse social behavior, supporting the interplay between the gut microbiota, the colonic and systemic immune systems, and social behavior.

## Methods

### Animals

Mice were housed in groups of five males or six females in a temperature (21 ± 2°C), humidity (55 ± 5%), and light-controlled (from 7 AM to 7 PM) room. Standard laboratory chow and water were available ad libitum. All experiments were approved by the Institutional Animal Care and Use Committee and the Israel Ministry of Health (Ariel University, Israel, protocol numbers: IL-181-08-19, and IL-226-07-21). Mice from generations 45–47 were used in this study. Each group of mice was grouped from at least two different cages to avoid the cage and litter effects.

### Dominant–submissive relationship (DSR) test

Dominant (Dom) and submissive (Sub) mice were selectively bred from outbred Sabra background strain mice (Envigo laboratories, Israel) based on their behavior in the DSR test. The DSR test is a food competition paradigm used to assess social interaction between pairs of mice as described previously [23]. This test was performed at each generation once mice reached the age of 8 weeks old. Briefly, pairs of mice of the same sex and similar weights (average 43.7 ± 2.1 g) were paired and tested according to the DSR protocol in the DSR apparatus (Additional file 1 S1A). Made up of Plexiglas, the DSR apparatus consisted of two identical chambers (12 cm × 8.5 cm × 7 cm) placed on opposite sides of the apparatus and connected by a tunnel (2.5 cm × 2.5 cm × 27 cm). In the center of the tunnel was a feeder tube with a 0.5-cm diameter hole in its bottom that provides sweetened milk (3% fat, 10% sugar); only one animal has accessed the feeder tube at any given time. On the tunnel, at the entrance to each chamber, gates prevented the mice from reaching the milk until they were removed, creating an equal starting point at the beginning of each session. Fourteen hours before each session, the mice were deprived of food, but water was provided ad libitum. A pair of mice were placed in the two separate chambers behind the gates on the test day. Mice were left in the chamber for 30 s for habituation. The gates were then removed, and for 5 min, the milk-drinking time was recorded manually for each mouse. DSR sessions were carried out for four consecutive days. A dominant–submissive relationship was determined if a significant difference (*p* < 0.05) was observed between the two mice’s daily drinking durations and if the difference in drinking scores was at least 40% (Additional file 1 S1B). In the DSR mouse model, more than 99% of the selectively bred Dom and Sub mice developed strong and stable DSRs inherited from their parents [23, 30]. Therefore, pups, younger than 8 weeks included in this study, although they have not been characterized by the DSR test, most likely possess a distinct Dominant or Submissive feature.

### Three-chamber sociability test (TCST)

The TCST was employed to assess the motivation of mice to socially interact with a stranger mouse [2]. A mouse was placed in the center chamber with free access to all chambers. On one of the two side chambers, an unfamiliar mouse of the same strain was caged inside a cylinder. An identical empty cylinder was placed in the opposite side chamber. The number of entries of the focal mouse into each of the side chambers was measured for 10 min. Social mice will prefer to spend significantly more time around the stranger mouse than in the empty chamber. Mouse movements were recorded using EthoVision 9.1 (Noldus, Netherlands) [2].

### Forced swim test (FST)

The FST was used for characterizing mice depressive-like behavior [31]. Mice were placed individually into an inescapable transparent glass cylinder (30 cm in height, 10 cm in diameter) filled 25‐cm high with water (25 ± 2°C). All animals were forced to swim for 6 min, during which immobility (floating in the water with only minor movements to keep afloat) was recorded and compared. Animals that failed to stay afloat were removed immediately. Each animal underwent the FST only once, after which they were dried with paper towels and placed in cages under a warm lamp for 10 min before being returned to their home cages**.**

### Elevated plus maze (EPM)

The EPM was used to assess mice emotional behavior by measuring general exploratory performance and avoidance of the aversive open arms of the maze [32]. Behavior in this task (i.e., activity in the open arms) reflects a conflict between rodent preference for protected areas (e.g., closed arms) and their innate motivation to explore novel environments. Arranged in a “+” shape, the EPM apparatus (54 cm in height and 66 cm in length) has two closed and two open arms, as well as an open center. A single mouse was placed in the center of the maze with its head directed toward a closed arm. The locomotory activity (distance traveled and speed) and exploratory behavior (the number of entries and the time spent in each arm) were recorded for 5 min using EthoVision 9.1 (Noldus, Netherlands). Anti-anxiety behavior was determined as increased time spent in the open arm and an increase in the ratio of open arm to closed arm entries.

### Fecal sample collection and microbiota DNA purification

Fecal samples were collected from 6 (3 male, 3 female) Dom and 6 Sub mice longitudinally (0, 1, 2, 3, 4, 8 weeks old) as described above, except for 1-week-old mice, which a part of their colon was collected for the analysis due to their small size. Genomic DNA was isolated using the ZymoBIOMICS DNA Miniprep Kit (Zymo Research, CA, USA) according to manufacturer instructions. DNA concentration and purity were quantified (Nanodrop 2000 Thermo Fisher Scientific, Waltham, MA, USA), followed by gel electrophoresis (1% agarose). The purified DNA samples were stored at − 20 °C for further analysis. DNA samples were sent to Hylabs (Hy Laboratories Ltd., Rehovot, Israel) for 16S sequencing, Set V3-V4: Forward primer (CS1_515F): ACACTGACGACATGGTTCTACAGTGCCAGCMGCCGCGGT, reverse primer (CS2_806R) TACGGTAGCAGAGACTTGGTCTGGACTACHVGGGTWTCT.

### Taxonomic analysis of Dom and Sub mice gut microbiota

FASTQ data were processed and analyzed using the QIIME2 pipeline, version 2023.5 [33]. Pair-end sequences were first demultiplexed using the q2‐demux plugin. To improve taxonomic resolution, reads were denoised and clustered using DADA2 via q2‐dada2 [34]. MAFFT [35] and fasttree2 [36] were used for alignment and phylogeny construction for all amplicon sequence variants (ASVs), using q2‐alignment and q2‐phylogeny plugins, respectively. Taxonomy classification was accomplished using a q2‐feature‐classifier [37], and final feature sequences were aligned against the Greengenes database (gg_12_10) with 99% confidence [38]. To avoid any possible contamination, the feature table was filtered with q2-feature-table. First, features that were annotated as mitochondria or chloroplast were filtered out. Next, features found in ≤ 10% per group from the total number of samples were removed, and features with < 0.001% frequencies in total were removed.

The analysis was performed on a rarefied table of 13,000 reads per sample. After sampling depth filtering in the alpha diversity, two Dom samples were filtered out. Alpha diversity was calculated using the Shannon vector diversity [39] measure, referring to the bacterial richness within the sample, and significant differences in bacterial richness between the groups were tested using the Kruskal–Wallis test. To evaluate if groups had significantly different bacterial communities, a permutational multivariate analysis of variance (PERMANOVA) was performed, as implemented in QIIME2 with the default of 999 permutations.

A principal coordinate analysis (PCoA) and heatmap plots were generated using “R” (https://www.r-project.org/) software (version 3.6.3). Beta diversity was assessed using a Bray–Curtis dissimilarity calculator (a commonly used Beta diversity index) and calculated using the ordinate function in the phyloseq package 1.30.0 [40]. The distance matrices were visualized by using PCoA. A heatmap was generated by calculating the standard deviation (SD) of the relative abundance of each taxon of all samples and plotting the 100 largest SDs in a heatmap.

Significant differences in bacterial genus-level abundance between the Dom and Sub mice groups were determined using linear discriminant analysis (LDA) of the effect size (LEfSe) with an LDA score higher than 2.0 and *α* values of 0.05 [41].

To identify common bacterial taxa associated with either Dom and Sub groups at week 1, we performed statistical analysis of zero-inflated count models; the zero-inflated negative binomial (ZINB) model in the R packages zinbwave [42] and scran, and then performed differential abundance testing using DESeq2 [43]. First, we calculated the weights using zinbwave (a general and flexible model for the analysis of high-dimensional zero-inflated count data). Then we estimated the size factors using DESeq2 and scran.

### Colon histology hematoxylin and eosin (H&E) and periodic acid–Schiff (PAS) staining

Colon specimens were collected from Dom (*n* = 32 males) and Sub mice (*n* = 29 males) at ages 1, 2, 3, and 12 weeks old, and from 12-week-old BS (*n* = 4 males) mice and incubated in 4% formalin for 24 h, after which the specimens were transferred to a 70% ethanol. The tissues were embedded in paraffin and sectioned into 4-μm sections. Slides were stained in H&E and PAS staining (Abcam, Cambridge, UK). PAS staining was done according to manufacture instruction and analyzed using ImageJ software [44].

### Fluorescent in-situ hybridization (FISH)

FISH analyses were used to quantify the distance of the colon bacteria from the epithelium and quantify the inner mucus layer length in the lumen. Tissue fixation (24–48 h) was performed using Carnoy fixative [60% (*v*/*v*) dry methanol, 30% (*v*/*v*) chloroform, 10% (*v*/*v*) glacial acetic acid]. After fixation, the tissue was washed twice in dry methanol for 30 min each, followed by two times in absolute ethanol for 20 min each, and incubated in two baths of xylene for 15 min each before paraffin embedding. Before rehydrating the slides, the tissues were incubated in washing (20 mM Tris-HCl, 0.9% NaCl; pH 7.4) and hybridization (20 mM Tris-HCl, 0.1% SDS; pH 7.4) buffers at 56°C overnight. Then slides were rehydrated and dipped in washing buffer at 56°C for 10 min. Probes (EUB338-1/2/3, for total of 16S bacterial staining) diluted in the hybridization buffer covered the slides with parafilm at 56°C overnight. The slides were stained for mucin using lectin (1:200, Vector Laboratories) for 2 h at room temperature in the dark. Hoechst (1:10, Vector Laboratories) was used to stain the nuclei. The results were viewed and analyzed with a fluorescent microscope using red, green, and blue channels (Zeiss).

### Gene expression quantification

RNA was extracted from the colon tissues of random Dom and Sub male mice (*n* = 5 from each group) longitudinally (0, 1, 2, 3, 4, 8, and 12 weeks old), using RNeasy Micro Kit (Qiagen, Germany). Reverse transcription was performed with a commercial cDNA Kit GoScript™ Reverse Transcriptase (Promega, Madison, WI, USA). RT-PCR was performed using a Fast SYBR® Green Master Mix (Applied Biosystems, MA, USA). The primers are presented in Table 1 (Hylabs, Rehovot, Israel). Reactions were performed using the QuantStudio1 96 RT-PCR System (Applied Biosystems, MA, USA).

**Table 1:** Primers used to detect gene expression in the RT-PCR

**Gene name**	**Forward**	**Reverse**
**HPRT**	TTGCGCTCATCTTAGGCTTT	TGTTGGATATGCCCTTG
**MUC 2**	TCCTGACCAAGAGCGAACAC	ACAGCACGACAGTCTTCAGG
**Cldn4**	AACTGCATGGAGGACGAGAC	GGGTTGTAGAAGTCGCGGAT
**Cldn7**	TGTCTTGTGGAGGGCTTGAG	TCCATCCAGAGCCCCTTGTA
**ZO3**	CCCCTTGTGATGAAAGCTGG	ATGGCCTCCAGAGACAGCTA

### Fluorescein-5-isothiocyanate (FITC)-dextran assay

To measure the gut permeability, we performed a FITC-dextran assay. Mice were fasted (no food or water) over night before the assay. Mice (weight 25–35 g) received an oral gavage of FITC-dextran (150 μL of 80 mg/ml FITC-dextran in PBS, Sigma Chemical), returned to their home cage, and provided food and water ad libitum. Mice were sacrificed 5–6 h after gavage, and cardiac blood was collected with a 1-ml syringe. Blood was incubated at room temperature for 30 min (protected from light) and then centrifuged for 20 min at 1500 × *g* at 4°C. Standards of FITC-dextran in PBS by dilution series: 8000, 4000, 2000, 1000, 500, 250, 125, and 0 ng/ml were prepared. Sera were diluted 1:1 in PBS. Mice sera and standards were dispensed in duplicates onto black 96-well plates and fluorescence imaged: excitation: 485 nm and emission: 528 nm in a microplate reader (Tecan).

### Lymphocyte isolation and flow cytometry

Colon tissues were treated with RPMI containing 1 mM DTT, 20 mM EDTA, and 2% FBS at 37°C for 10 min to remove epithelial cells and then minced and dissociated in collagenase solution (1.5 mg/ml collagenase II (Gibco, Thermofisher, USA), 0.5 mg/ml dispase (Gibco, Thermofisher, USA) and 1% FBS in RPMI) with constant stirring at 37°C for 30 min. Single-cell suspensions were then filtered and washed with 4% RPMI solution and stained with antibodies against mouse CD45, CD19, CD4, CD8, Foxp3, Helios, and Rorγ (Miltenyi biotec) (Table 2). For intracellular staining of transcription factors, cells were stained for surface markers and fixed in eBioscience Fix/Perm buffer (Thermofisher) overnight, followed by permeabilization procedure (Thermofisher) for 45 min in the presence of antibodies. Cells were analyzed with a BD LSRFortessa flow cytometer (BD, USA) and data processed with FlowJo software (BD Life Sciences).

**Table 2:** List of antibodies used in the FACS experiments

**Antibody**	**Fluorophore**	**Company**
**CD45**	FITC	Miltenyi Biotec
**CD19**	PerCP-Vio600	Miltenyi Biotec
**CD4**	Vio-blue	Miltenyi Biotec
**CD8**	Vio-green	Miltenyi Biotec
**Foxp3**	APC	Miltenyi Biotec
**Helios**	PE-Vio615	Miltenyi Biotec
**Rorγ**	PE	Miltenyi Biotec

### Fecal transplantation experiment

Male GF Swiss Webster mice were inbred and housed in semi-solid GF isolators (3–5 mice per cage) at the Azrieli Faculty of Medicine (Safed, Bar Ilan University, Israel) under a 12-h light/12-h dark cycle, at 22 °C, with autoclaved food and water available ad libitum. Fecal transplantations were performed in 8–10-week-old GF mice (*n* = 17). Fresh fecal samples were collected and pooled for transplantation from adult (12 weeks) donor Dom (*n* = 4) or Sub (*n* = 4) mice. Donor mice were housed in individual cages together with four other mice (Dom or Sub) that were not used for this experiment (in our mice facility, we house five mice per cage). The stools from four mice (per each behavioral phenotype) were collected into one tube that served as the pooled fecal material for transplantation to each GF group (GF/Dom and GF/Sub). After the FMT experiment, the transplanted GF mice were housed as follows (GF/Dom: four and three per cage and GF/Sub: four and three per cage, GF/Con: three in one cage). Mouse inoculation of the respective fecal material (Dom/Sub/Con, 200 µl) was performed by oral gavage using a sterile feeding tube (20 ga × 38 mm, Instech Laboratories, Inc., Plymouth Meeting, PA) after suspending each stool pellet in 1 ml of sterile PBS. The effect of fecal transplantation on GF mouse colon SCFAs was assessed. Transplanted GF mice behavior were assessed using three-chamber sociability test and Forced swim test and were published previously [28].

### Fecal sample collection and short-chain fatty acid quantification

Fresh fecal samples (~ 250 mg each) were collected from 3-month-old Dom (*n* = 8) and Sub (*n* = 7) mice. Samples were collected from 9:00 AM to 12:00 AM by placing mice individually in sterile cages and retrieving the feces using sterile forceps. Samples were placed in pre-weighed sterile tubes and immediately stored at − 80°C. The targeted metabolomics was performed at the Weizmann Institute, Israel, using LC-MS analysis to detect butyrate, propionate, and acetate SCFAs.

### In vivo therapeutic evaluation

The therapeutic effects of an anti-inflammatory (AI) agent and a gut permeability reducing agent were evaluated in Sub mice. Briefly, 40 Sub male mice were divided randomly into four groups (*n* = 10) as follows: control group, that received normal drinking water daily (P/O); AI group—mice were orally administered with celecoxib (Cox-2 inhibitor, 16 mg/Kg) (Trima, Israel) daily for 4 weeks; hyaluronic acid (HA) group—mice were orally administered with HA (30 mg/kg) (low Mw HA, R&D Systems, Minneapolis, MN, United States), once a week for 6 weeks; and a combination treatment group that were orally administered with a combination of the two agents. To ensure accurate dosing, oral gavage administration was used. Behavioral assessment of all mice (using TCST, EPM and FST) was performed during 4 to 6 weeks of treatment, and gut permeability assessment using the FITC-dextran assay was performed after 6 weeks of treatment.

### Cytokine profiles in colon tissues

Cytokine differences in the colon tissue of control and treated Sub mice, at 4-month-old, were assessed by an antibody-based protein array (Proteome Profiler: Mouse Cytokine Array (R&D Systems, Minneapolis, MN, USA)), according to the manufacturer’s instructions. We used a pooled protein extract from colon tissues removed from three random mice from each group. The average signal of pixel density from duplicate cytokines was determined using ImageQuant TL software. The relative intensity of the reference values (three inside control duplicates in each membrane) was included with densitometry calculations ImageQuant TL software.

### Statistical analyses

All statistical analyses were performed using GraphPad Prism 6 unless otherwise noted. Quantitative results are expressed as means ± SD and were analyzed using a student’s *t*-test for individual comparisons or one- or two-way ANOVA, followed by a Bonferroni means separation test for multiple comparisons. The statistical significance of differences between groups is presented graphically as (*) for *p* < 0.05, (**) for *p* < 0.01, and (***) for *p* < 0.001.

## Results

### Dom and Sub mice gut microbiota compositions differ from early infancy

**Fig. 1:**
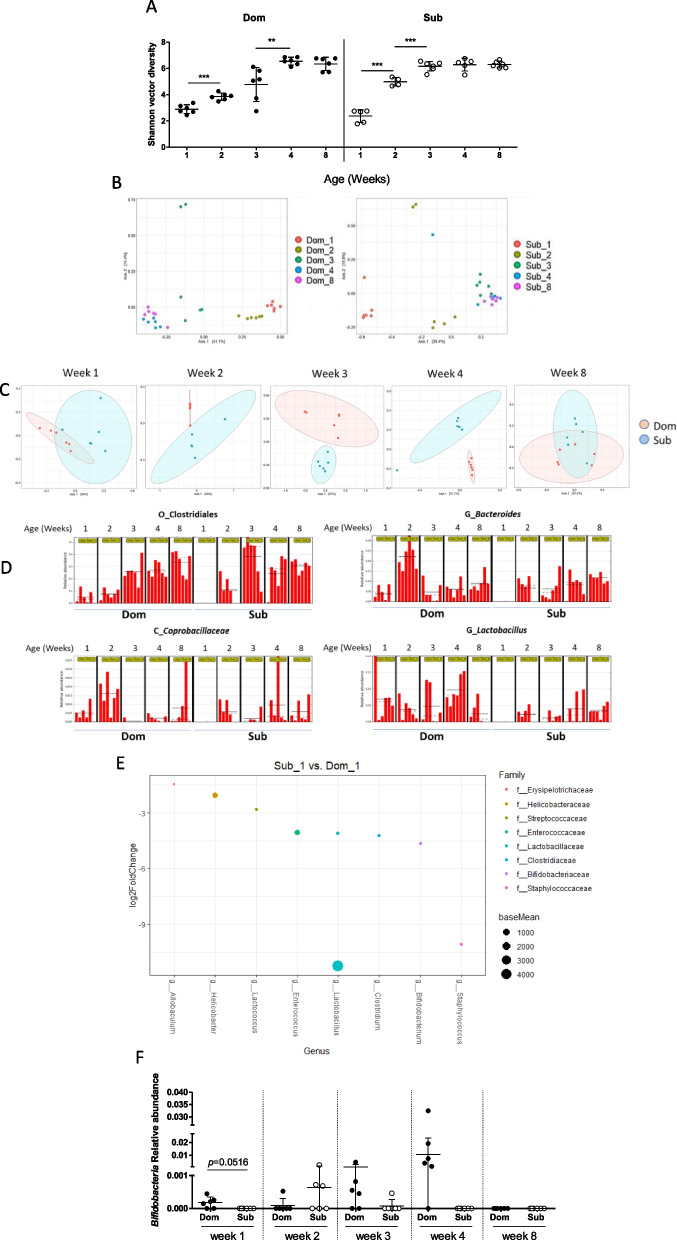
Dom and Sub gut microbiota compositions differ from early infancy to adulthood. **A** Alpha diversity of the gut microbiotas of Dom and Sub mice at different ages, from first week (Dom 1 or Sub 1) to eight-weeks (Dom 8 or Sub 8), *n* = 6 (3 males and 3 females) in each group. **B** PCoA of Dom and Sub mice at different ages. **C** PCoA of Dom vs. Sub mice at different ages. **D** Abundance histograms of *Clostridiales*, *Coprobacillaceae*, *Bacteroides*, and *Lactobacillus* generated using LEfSe with collapsed features. **E** Differential abundance analysis of ASVs that had a significantly altered abundance in Dom and Sub at the first week using DESeq2 analysis. **F** The relative abundance of the genus *Bifidobacterium* of Dom and Sub gut microbiotas in mice aged one to eight- weeks. (**) *p* < 0.01 (***), *p* < 0.001. Error bars show standard deviation

Dom and Sub mice exhibit significantly different sex-independent gut microbiota compositions in adulthood [45]. To determine the gut microbiota dynamics, we performed a longitudinal gut microbiota characterization. We found that Dom and Sub mice showed a significant and continuous increase in gut microbiota alpha diversity from 1 to 8 weeks old (Fig. 1A), as expected [46]. Alpha diversity indices of Observed ASVs, and PD, the Faith’s phylogenetic diversity demonstrated a similar pattern (Additional file 1 S2). The beta diversity between Dom and Sub mice gut microbiotas at each age was also significantly different, as demonstrated with PERMANOVAs on weighted distance metrics (Fig. 1B). The differences in beta diversity between the mouse phenotypes were greater at the age of 1 to 4 weeks (*p* < 0.01), and these pronounced differences were reduced at 8 weeks old, although the groups’ microbiotas still cluster separately (Fig. 1C, *p* > 0.05). The same trend is demonstrated in the heatmaps (Additional file 1 Fig. S3). Noticeably, the gut microbiota composition of Dom and Sub mice evolved until the age of 8 weeks, but the Sub mouse gut microbiota composition partially stabilized at an earlier age. Sub mice gut microbiota composition changed from four to eight weeks old (*p* = 0.02), but not as significantly as of Dom mice (*p* = 0.007, Fig. 1B and Additional file 1 S3).

LEfSe analysis which uses collapsed features revealed different biomarkers at each age (Fig. 1D). Several key developmental bacteria [47, 48] were detected only in Dom mice at 1 week old and detected in Sub microbiotas a week later, at the age of 2 weeks. These unique bacteria belonged to the *Clostridiales* order (Fig. 1D, Dom vs. Sub—mean relative abundance ± SD—0.05 ± 0.04 vs. 0 ± 0, respectively), the *Coprobacillaceae* family (Fig. 1D, Dom vs. Sub mean relative abundance—0.0050 ± 0.004 vs. 0 ± 0, respectively), and the *Bacteroides* and *Lactobacillus* genera (Fig. 1D, Dom vs. Sub mean relative abundance 0.04 ± 0.03 vs. 0.0001 ± 0.0001 and 0.069 ± 0.06 vs. 0 ± 0, respectively). To find altered ASVs without collapsing the features, DESeq2 analysis was used. Moreover, because zero-inflated data can lead to increased estimates of variance, zero-inflated count models appear to provide a better fit for 16S datasets displaying a bimodal distribution (i.e., point mass at zero and second mass separate from zero) [42, 49]. Thus, we also confirmed our results using the R packages zinbwave (a zero-inflated count model—zero-inflated negative binomial (ZINB) model) and scran, followed by differential abundance testing analysis using DESeq2 (Fig. 1E). This analysis identified nine significantly different features between Dom and Sub mice at the age of 1 week (corrected *p*-value ranging from 7.01e − 19 to 2.64e − 02). All these ASVs exhibited decreased levels in Sub mice (log2 fold change—range: − 11.2 to − 1.4, mean log2 fold change: − 4.97, Fig. 1E).

Moreover, using DESeq2 analysis [50], *Bifidobacterium pseudolongum*, a key species in maternal milk sugars digestion and immune regulation [51], was found to be differentially abundant between the groups at the age of 1 week, present only in Dom mouse microbiotas. At the ages of 2, 3, and 4 weeks, the prevalence of this bacterium varies in both mouse phenotypes without significant differences (Fig. 1F, *p* > 0.05). Overall, longitudinal gut microbiota analyses indicate that Dom and Sub mice possess significantly different gut microbiota patterns from early infancy.

### Age-dependent eWAT and gut tissue growth patterns differ between Dom and Sub mice

**Fig. 2:**
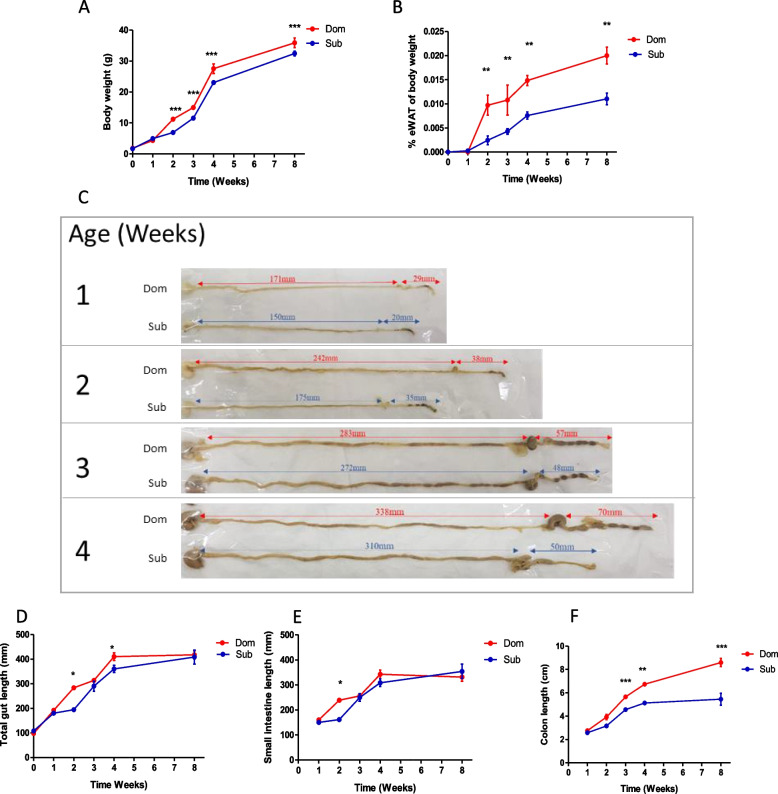
Dom and Sub mice differ in body weight, eWAT content, and colon development from early infancy to adulthood. **A** Body-weight follow-up of Dom (*n* = 90; 45 males) and Sub (*n* = 92; 42 males) mice. **B** eWAT mass (normalized to body weight) follow-up of Dom and Sub male mice. **C** Visualization of eight representative mouse intestines removed from Dom and Sub male mice. Colon length (in millimeters, mm) of Dom vs. Sub at one to four-weeks of age; means are 29 vs. 20, 38 vs. 35, 57 vs. 48, and 70 vs. 50 mm, respectively. **D** Complete intestine length follow-up. **E** Small intestine length follow-up. **F** Colon length follow-up. eWAT and colon tissue follow-up was performed on the same mice (Dom, *n* = 53; Sub, *n* = 51). Statistical significance was determined using a student’s *t*-test, (*) *p* < 0.05, (**) *p* < 0.01, (***) *p* < 0.001. Error bars show standard deviation

Consistent with our previous findings [28], Dom mice weigh significantly more than Sub mice from the second week of life (Fig. 2A), in spite similar food intake of both mice phenotypes measured in adulthood [28]. These body weight differences are presumably derived from the early infancy gut microbiota differences and smaller epididymis white adipose tissue (eWAT) mass. A longitudinal eWAT and gastrointestinal (GI) tract comparative development analysis revealed an earlier eWAT tissue development in male Dom mouse compared to Sub mice; (3.9-fold increase in eWAT tissue, already at the age of 2 weeks where at this age, the eWAT of Sub mice was barely detectable (Fig. 2B, *p* < 0.001). Moreover, although Sub male mice started to develop high eWAT mass at the age of 3 weeks and thereafter, it was continually significantly lower than that of Dom mice (*p* < 0.01).

Additional analyses revealed similar mass of the spleen and liver tissues in Dom and Sub mice, at the different ages tested (Additional file 1 Fig. S4). Length measurements of the GI showed that the entire gut length of Dom and Sub mice was similar and only differed at the age of 2 and 4 weeks (Fig. 2C-D). Detailed analyzes of the gut parts revealed that although Dom and Sub mice small intestine lengths were similar (*p* > 0.05, except at the age of 2 weeks, Fig. 2E), a dramatic sex-independent colon length reduction was observed in Sub mice compared to Dom mice, from the third week of life (Fig. 2F, *p* < 0.001).

### Sub mice exhibit higher mucin expression and age-dependent elevated gut permeability

**Fig. 3:**
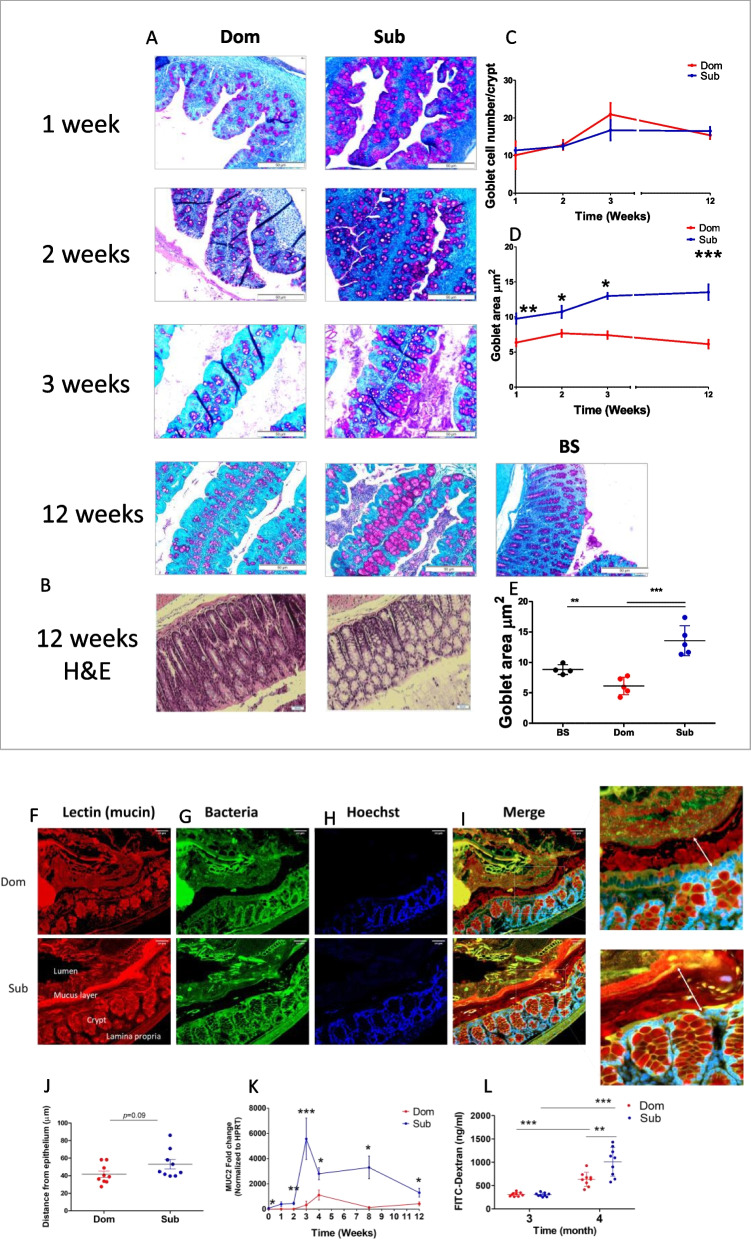
Sub mice compared to Dom mice possess age-dependent higher mucin expression and increased gut permeability. **A** PAS staining of Dom (*n* = 32 males) and Sub mice (*n* = 29 males) at ages one, two, three, and 12-week-old and 12-week-old BS mice (*n* = 4 males). **B** H&E staining of 12-week-old Dom (*n* = 10 males) and Sub mice (*n* = 10 males). **C** Quantification of the goblet cell number according to the mucin droplets per crypt from 10 fields of each mouse at ages 1, 2, 3, 4, and 12 weeks old. **D** Quantification of the goblet cell area (μm^2^) according to the mucin droplets from 10 fields of each mouse at ages 1, 2, 3, 4, and 12 weeks old. **E** Quantification of the goblet cell area (μm^2^) according to the mucin droplets from ten fields of Dom, Sub, and BS mice at the age of 12 weeks. **F** Dom and Sub mouse colon mucin staining using lectin labeling. **G** Dom and Sub mouse colon bacteria staining using FISH probes. **H** Dom and Sub mouse colon nuclei staining using Hoechst labeling. **I** Merged staining. **J** Quantification of the distance between the lumen bacteria and the colon epithelium based on the FISH staining (panels **F**–**I**), using the Zen 3.4 software. White arrows define the distance measured. **K** MUC2 gene expression normalized to HPRT in Dom (*n* = 30 males) and Sub mice (*n* = 34 males), at the ages of 0, 1, 2, 3, 4, 8, and 12 weeks old. **L** Gut permeability of Dom and Sub mice (*n* = 9 males in each group, in ng/ml), at the ages of three and four months old, based on the detection of serum FITC-dextran levels, 5–6 h post FITC-dextran oral gavage administration. Statistical significance was determined using a student’s *t*-test; (*) *p* < 0.05, (**) *p* < 0.01, (***) *p* < 0.001. Error bars show standard deviation

To characterize the differences in Dom and Sub mice colon physiology, we evaluated the colon mucus layer and determined their gut permeability. Total mucin analysis of colon tissues, performed by periodic acid–Schiff (PAS) staining (Fig. 3A), demonstrated that while the number of mucin-producing goblet cells per crypt was similar between Dom and Sub mice in all the tested ages (Fig. 3C), the mucin vesicle area from early infancy was significantly higher in Sub mice compared to Dom mice and increased significantly with age (Fig. 3D, *p* < 0.01). To determine if the mucus layer thickness is increased in Sub mice, or decreased in Dom mice, we compared these findings to the mucin levels in adult Sabra background strain (BS) mice at 12 weeks old, the age at which we observed the highest difference in mucin levels between Dom and Sub mice. Indeed, at this age, the colon mucin area of Dom and BS mice was similar, while Sub mice colon tissue exhibited a higher mucin area (Fig. 3E, *p* < 0.01), suggesting that Sub mice are mucin hyper-producers.

To further evaluate the mucin structure, using fluorescence in situ hybridization (FISH), we analyzed the distance from the inner mucus layer to outside the lumen epithelium in adult Dom and Sub mice (Fig. 3F–I). Quantification of the average distance between the bacteria and the epithelium indicated that in Sub mice colons, the bacteria are more distant from the mucin epithelium layer than in Dom mice (Fig. 3J, mean ± SD, 50 μm ± 15.1 in Sub mice versus 40 ± 10.4 μm in Dom mice, *p* = 0.09). Although this difference was statistically insignificant, this 10-μm difference in bacterial mucus layer distance is in line with the heavier mucus layer that characterizes the Sub colons. It was highly noticeable that Sub mouse mucin was great than that of Dom mice at three-month-old at the lumen area, which was not shown in the PAS staining due to the hydrophilic formalin that washed the lumen mucin away during the tissue fixation (Fig. 2A).

To further confirm mucin hyper-production in Sub mouse, we determined the expression levels of MUC2, the most abundant peptide in the colon mucin [52] by RT-PCR, and discovered significantly higher expression in Sub mouse colons than in Dom mice, from birth to adulthood, at all the ages tested (Fig. 3K). Of note is that at the age of three weeks, when Sub mice MUC2 expression was the highest, the differences in colon length between Dom and Sub mice also became significant.

Our results revealed that Sub mice colons are significantly shorter than Dom mice colons (Fig. 2), which possibly suggest an inflammatory process [53]. Therefore, we sought to evaluate and compare the colon function in both mouse phenotypes by determining their gut permeability. Using a FITC-dextran assay, which measures the sera level of labeled dextran leaked from the gut due to enhanced gut permeability, we demonstrated that at the age of 3 months, Sub and Dom mice had similar gut permeability; however, at the age of four-months, Sub mice possess age-dependent increased gut permeability (Fig. 3L).

### Sub mice exhibit lower colonic Tregs in the spleen and colon compared to Dom mice

**Fig. 4:**
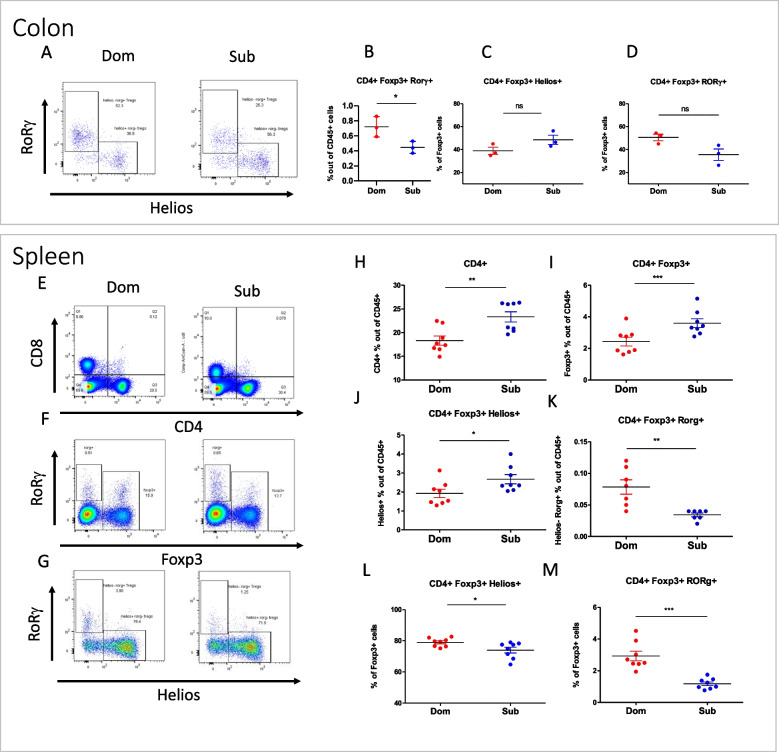
Adult Sub mice demonstrate lower colon and spleen Treg cell levels compared to Dom mice. Frequencies of Foxp3+/Rorγ+ Tregs among total Tregs in colon and spleen tissues of Dom and Sub mice were determined by flow cytometry. **A** Representative FACS plots depict Helios/Rorγ+ expression by Tregs from Dom and Sub mice (*n* = 6). **B** Tregs were analyzed for frequencies of CD19-/CD4+/Foxp3+/Rorγ+ Tregs among CD45+ cells. **C** Tregs were analyzed for frequencies of Foxp3+/Helios+ Tregs among CD4+ cells. **D** Tregs were analyzed for frequencies of Foxp3+/Rorγ+ Treg among CD4+ cells. **E** Representative FACS plots depicting CD8+/CD4+ expression by T cells from the spleens of Dom and Sub mice. **F** Representative FACS plots depicting Foxp3+/Rorγ+ expression by CD4+ T cells from the spleens of Dom and Sub mice. **G** Representative FACS plots depicting Helios+/Rorγ+ expression by Treg from the spleen of Dom and Sub mice. **H** T cells were analyzed for frequencies of CD19-/CD4+ T cells among CD45+ cells. **I** T cells were analyzed for frequencies of CD19-/CD4+/Foxp3+ Treg cells among CD45+ cells. **J** Treg cells were analyzed for frequencies of CD19-/CD4+/Foxp3+/Helios+ Treg cells among CD45+ cells. **K** Treg cells were analyzed for CD19-/CD4+/Foxp3+/Rorγ+ Treg cells frequencies among CD45+ cells. **L** Treg cells were analyzed for frequencies of Foxp3+/Helios+ Treg cells among CD4+ cells. **M** Treg cells were analyzed for frequencies of Foxp3+/Rorγ+ Treg cells among CD4+ cells. (*) *p* < 0.05 (**) *p* < 0.01, and (***) *p* < 0.001, analyzed by Student’s *t*-test. Error bars show standard deviation. ns nonsignificant

Sub mice shorter colons and increased gut permeability, both of which are signs of inflammation [53], led us to perform antibody-based FACS analysis of Dom and Sub mice colon and spleen immune cells. The strategy we used for the immune cell population analysis is presented in Additional file 1 Fig. S5 and we focused on colonic Tregs, which are highly affected by the gut microbiota [20]. Colon immune cell (lamina propria lymphocyte) analysis revealed that Sub mice contained 61% less colonic Tregs (defined as CD19-CD4 + FoxP3 + Rorγ + Helios-) [54] than Dom mice (Fig. 4B, *p* < 0.05).

The spleen immune cells of Sub mice exhibited a 27% increase in CD4+ cells compared to Dom mice (Fig. 4H, *p* < 0.01). In addition, we found an increase of 47% in total Tregs (CD19-CD4 + FoxP3 +) and a 38% increase in thymic Tregs (CD19-CD4 + Foxp3 + Helios + , tTreg) in Sub mice compared to Dom mice (Fig. 4I-J, *p* < 0.01). In contrast to these findings, Sub mice exhibited a 75% decrease in splenic CD4 + Foxp3 + Rorγ + Tregs compared to Dom mice (Fig. 4K, *p* < 0.01). Furthermore, we characterized the different Treg types out of the total Tregs (Foxp3 + cells), and we found that both tTreg (CD19-CD4 + Foxp3 + Helios +) and colonic Treg (CD19-CD4 + Foxp3 + Rorγ +) levels were lower in Sub mice compared to Dom mice (Fig. 4L and M). These combined results suggest that Sub mice may exhibit unbalanced colonic immune activity and inflammation due to the unbalanced Treg populations found.

### Sub mice possess lower gut short-chain fatty acids levels

**Fig. 5:**
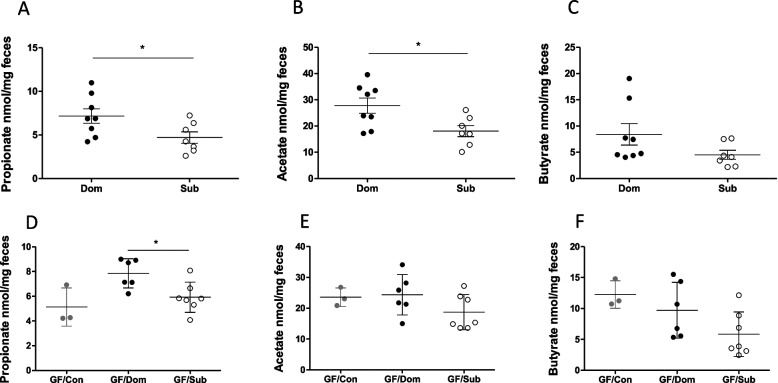
Adult Sub mice possess microbiota-induced lower fecal SCFA levels compared to Dom mice. **A** Propionate, **B** acetate, and **C** butyrate concentrations (nmol/mg feces) in Dom (*n* = 8) and Sub (*n* = 7) stools. **D** Propionate, **E** acetate, and **F** butyrate concentrations (nmol/mg feces) in 2.5 months transplanted GF mice. Transplants were of PBS (*n* = 3) or stools from Dom (*n* = 6) or Sub (*n* = 7) mice resuspended in PBS. Statistical significance was determined using a student’s *t*-test and one-way ANOVA, (*) *p* < 0.05. Error bars show standard deviation

The reduction in colonic Tregs together with increased gut permeability and an altered gut microbiota in Sub mice led us to hypothesize that their gut SCFA contents would differ from Dom mice. We performed targeted metabolomics on adult eight-week-old Dom and Sub mice to quantify the three primary SCFAs which regulate Treg activity and tight junction expression in the colon: propionate, acetate, and butyrate [20]. Fecal metabolomic analysis revealed that Sub mice possessed a 52% and 53% decrease in propionate and acetate, respectively (Fig. 5A and B, *p* < 0.05) and showed a tendency toward lower butyrate levels (Fig. 5C; 1.8-fold compared to Dom mice, *p* > 0.05). A similar pattern was observed in fecal-transplanted GF mice, with a significant 32% reduction in propionate in Sub-transplanted GF mice compared to Dom-transplanted GF mice (Fig. 5D), suggesting that the decreased propionate levels in Sub mice may result from their altered gut microbiota composition.

### In-vivo therapeutics with hyaluronic acid or celecoxib improved Sub mouse social behavior and reduced the inflamed colon features

**Fig. 6:**
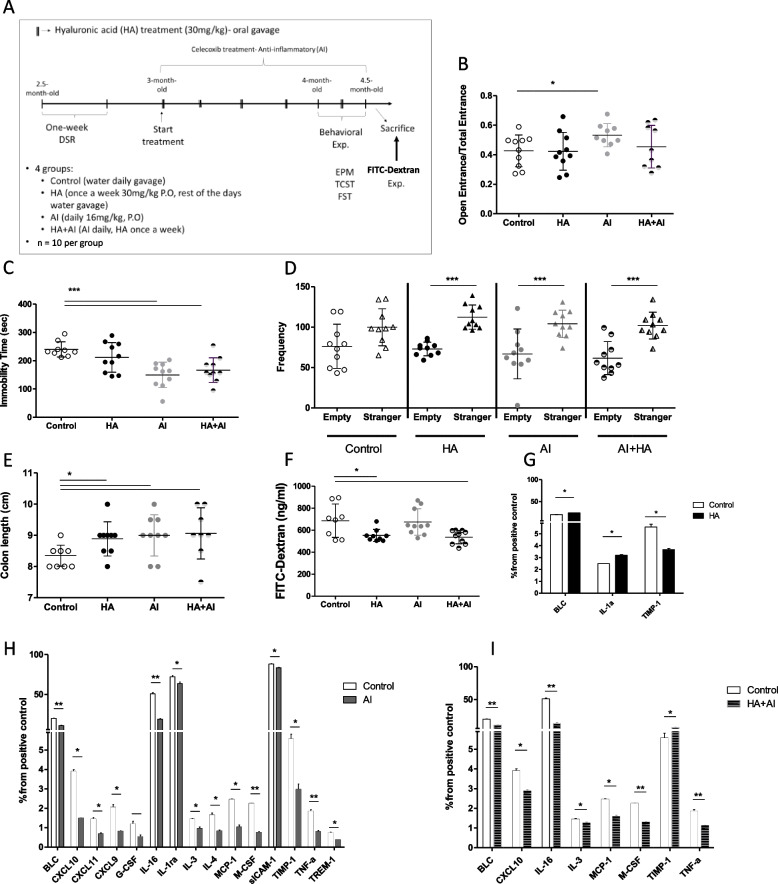
Anti-inflammatory and gut permeability reduction treatments modulated Sub mouse behavior, colon length and inflammation, and gut permeability. **A** The study design of the treatment experiment performed on 40 mice (4 groups of 10 mice each—control (water-treated), HA-treated, AI-treated, and HA + AI-treated). **B** EPM test of Sub (*n* = 40 males, 10 in each group) controls and mice treated with HA, AI, and HA + AI agents. *Y*-axis shows the frequency of entering the open arms normalized to the frequency of entering the open and close arms. **C** FST of Sub mice treated with HA, AI, and HA + AI agents and controls. *Y*-axis shows the time mice were immobile in the water. **D** TCST of Sub mice treated with HA, AI, and HA + AI agents and controls. *Y*-axis shows the nose-point frequency to enter the area around the stranger mouse. **E** Colon length of Sub mice treated with HA, AI, and HA + AI agents and Controls. **F** FITC-dextran assay of gut permeability, *Y*-axis shows FITC-dextran concentration in the mouse serum (ng/ml) 5 h after oral gavage at 4.5 months old. **G**–**I** A cytokine array comparison of pooled proteins extracted from the colons of Sub mice: controls (*n* = 3) and those treated with **G** HA (*n* = 3), **H** AI, and **I** HA + AI. Panels **G**, **H**, and **I** present only the colon cytokines with significantly different expression per treatment, compared to the Sub control group. Each bar represents the average duplicate cytokine expression normalized to the positive control. Control Sub mice demonstrated a significantly higher cytokine level than AI-treated Sub mice. Statistical significance was determined using a Student’s *t*-test, (*) *p* < 0.05, (**) *p* < 0.01, and (***) *p* < 0.001. Error bars show standard deviation. AI anti-inflammatory, HA hyaluronic acid, EPM elevated plus maze, TCT three chambers test, FST forced swim test

Considering the systemic inflammation of Sub mouse previously reported [27], the inflamed colons and dysregulated gut permeability we discovered, we sought to examine the effect of two agents on adult (2.5 months old) Sub mice. The treatment regimens included (i) a gut permeability reduction agent hyaluronic acid (HA) [29], (ii) an anti-inflammatory (AI) agent—celecoxib (Cox-2 inhibitor) [55], and (iii) a combined treatment of the two agents (Fig. 6A). Following the indicated treatment modalities, we performed EPM, TCST, and FST behavioral paradigms to examine treatment-influenced behavioral changes of Sub mice. The order of the sequential behavioral tests was set to minimize the stress effects, as EPM is the least stressful and FST is the most. Moreover, to reduce the stress between the tests, the mice rested for at least 4 days between each test. Sub mice treated with the celecoxib exhibited a significant decrease in their anxiety- and depressive-like behaviors (Fig. 6B-C, 24% and 66%, respectively). Moreover, they were significantly more sociable than the control Sub mice treated with saline (Fig. 6D). In addition, treating mice with celecoxib significantly increased their colon length compared to the control group (Fig. 6E). These results support the strong relation between Sub mice’s inflammatory state and their behavioral deficits. To examine whether their inflammation state originated from their increased gut permeability, we treated Sub mice with HA, an agent known to increase colonic tight junction protein expression [29]. HA-treated Sub mice demonstrated significantly decreased gut permeability (Fig. 6F, 25%), 6% increase in colon length (Fig. 6E), and improved social behavior (Fig. 6D). Moreover, a colon cytokine array (Additional file 1 Fig. S6) demonstrated a significant decrease in 15 pro-inflammatory cytokines after celecoxib treatment (Fig. 6H) and a decrease in seven pro-inflammatory cytokines after a combination treatment of HA and AI (Fig. 6I). After HA treatment, Sub mice exhibited similar colon cytokine profiles with changes only in three cytokine levels (Fig. 6G). Both the treatments of HA and AI exhibited negligible impact on the composition of the gut microbiome (Additional file 1 Fig. S7). Overall, intervention with both agents improved the inflammatory status of Sub mice colons and significantly promoted the social behavior deficits of Sub mice.

## Discussion

Our data demonstrate that social behavior correlates with early-infancy gut microbiota composition, colon anatomy, gut permeability, SCFA levels as well as colonic and systemic inflammatory states. Importantly, these findings were supported by treating Sub mice with hyaluronic acid, which reversed the elevated gut permeability, substantially improving their sociability.

Our previous research proved that social behavior patterns exhibited by adult Dom and Sub mice are shaped by their gut microbiota [45]. This study reveals that these differences are already present in early infancy and are reflected by a reduction or absence of critical bacteria in Sub mice, including Clostridiales, *Lactobacillus,* and* Bifidobacterium* [56]. In line with our findings, reduction of the order Clostridiales, which contributes immensely to SCFAs production in the gut [57], was reported in various psychiatric diseases, including depression and schizophrenia [58]. The *Lactobacillus* genus was shown previously to reduce adverse effects of stress in rodents [59]. More specifically, *Lactobacillus rhamnosus JB-1* reduces stress-induced immobility duration [60] and anxiety-like behaviors [61] in adult male mice. In infants, *Lactobacillus casei* GG increased epithelial barrier function [62] and reduced gut permeability [63]. *Bifidobacterium*, a widely recognized probiotic bacterial genus [47], was reported to favor behavior and immunity. For example, *Bifidobacterium breve CCFM1025* was shown to reverse chronic stress-induced depressive symptoms and gut microbial abnormalities in mice [64]. In another study, *Bifidobacterium breve MCC-117* colonization in infants was reported to initiate the development and maturation of the regular immune system by stimulating dendritic cells through Toll-like receptors [65]. Overall, these bacteria reduce inflammation in the developing colon; thus, their absence in Sub mice microbiota may explain their inflamed colon.

Apart from the variations in Dom and Sub mouse gut microbiota establishment, throughout their maturation and adulthood [28], Sub mice exhibited substantial alterations in colon morphology, reduced colon length, thicker mucin layer, and lower Treg cells and SCFAs than Dom mice. Numerous works demonstrate a link between colon shortening and GI inflammation [66–68]. Thus, we speculate that the short colon we observed in Sub mice results from chronic inflammation, as supported by other experiments in our study. Moreover, mucin secretion is modulated by bacterial factors [69]. Hence, the thickened colon mucin layer and the elevated MUC2 expression observed in Sub mice could be explained as a compensation mechanism for their altered gut microbiota composition [28]. Similarly, there is evidence that gut dysbiosis (GF conditions, absence of specific taxa) can imbalance mucus production and degradation, resulting in the thickening of the mucus layer [70, 71]. Further, it has also been demonstrated that stress can shift the O-glycosylation patterns of mucins in rats [72]; this shift can be prevented by probiotic treatment [72]. Thus, we can speculate that the inherited stress vulnerability of Sub mice is in a crosstalk with the altered gut microbiota, which then leads to increased colon mucin. A longitudinal comparison of the tight junction expression levels between Dom and Sub mice colons demonstrated higher expression levels of Claudin-7 (CLD7), Claudin-4 (CLD4), and ZO3 in Sub mice from birth to adulthood compared to Dom mice, like MUC2 expression levels pattern. Also, their expression levels in Sub colons varied compared to their expression levels in Dom colons, which stayed stable throughout their maturation (Additional file 1 Fig. S8). The tight junction overexpression may also be a compensation mechanism in Sub mice, similar to mucin hypersecretion. The colon inflammation observed in Sub mice can be explained by the reduction in Tregs, known to increase inflammation [73]. Moreover, Tregs may be protective against depressive-like behavior [73, 74]. The altered Sub mouse colon immunity could result from the reduced *Lactobacillus*, *Bifidobacterium,* and Clostridiales, all responsible for immune maturation and balanced inflammation. Finally, The SCFAs propionate and acetate, which were reduced in Sub mice are the primary metabolites produced in the colon by bacterial fermentation of dietary fibers and resistant starch and are known to have protective effects [75]. Administration of sodium propionate was shown to induce antidepressant-like effects in rats [76] and acetate treatment of microglia primary culture reduced inflammatory signaling [77]. These metabolites could be the mediators of the gut microbiota, the immune cells, and the nervous system. Interestingly, the genus *Prevotella*, a propionate-producer in the gut [78], was previously demonstrated to be significantly decreased (27-fold, *p* < 0.05) in adult Sub mice [28]. The multi-aspect colon physiology dysregulation can potentially represent or cause Sub mouse colon inflammation.

Treating adult Sub mice orally with celecoxib, a cyclooxygenase-2 (Cox-2) inhibitor, reduced the colon cytokines levels, significantly improved social interactions, and decreased depressive-like behavior. Moreover, treating mice only with HA similarly improved social behavior via a significant reduction in gut permeability that presumably restricted the inflammation to the colon. These successful treatments outcomes suggested that therapy with these two drugs could mostly recover Sub colon inflammation status, positively affecting behavior. The use of HA to treat an inflamed colon was previously described in a study that used hyaluronic acid–bilirubin nanomedicine and successfully restored the epithelium barriers in a murine model of acute colitis [79]. However, the use of HA in treating inflamed-colon-induced social behavior deficits was first described herein.

Overall, data from this study suggests that the behavioral deficits typical of Sub mice may result from inflammation, partly from the colon, and may be present early in infancy. Therapeutics were also accompanied by increased colon length, suggesting a direct connection between social behavior and colon inflammation. Currently, research on the correlation between gut-derived bacterial-induced inflammation and decreased social exploration relies on lipopolysaccharide (LPS)-induced inflammation in experimental models in mice [80] and rats [81]. Similar to our study, peripheral administration of Cox inhibitors blocked LPS-induced suppression of social behavior in adult mice [82]. The data presented in this current study is based on an animal model. Therefore, future clinical studies are warranted to confirm the positive therapeutic effects of these agents on social behavior deficits.

## Conclusions

**Fig. 7:**
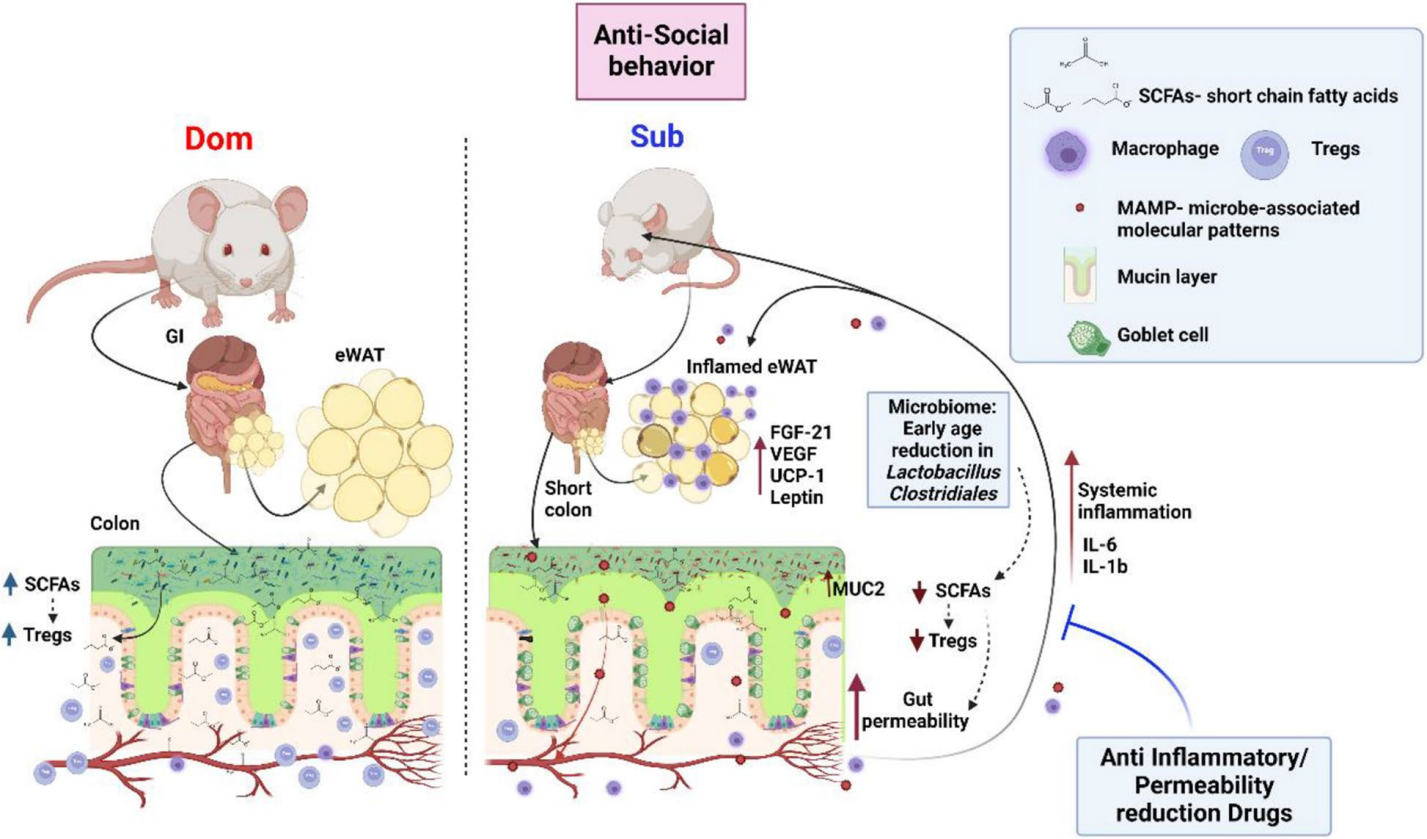
A model of the Sub mouse altered gut–brain–axis crosstalk that shapes anti-social behavior. Sub mice are born with lower gut microbiota diversity and lack *Lactobacillus* and *Clostridiales* that are critical bacteria essential for tissue development and function. The altered microbiota is, in part, characterized by reduced abundance of SCFA-producing bacteria which leads to increased mucin secretion reflected by dramatic elevation in MUC2 gene expression, and altered immunity, reflected by lower Treg cells, leading to imbalanced inflammation. The exacerbated colon inflammation causes increased gut permeability and systemic inflammation primarily due to the unregulated transfer of bacterial substances to the circulating blood. The systemic inflammation may affect the eWAT metabolic and inflammatory profile, induce neuroinflammation, and affect Sub mice behavior

To conclude, we propose a pathway (Fig. 7) in which the gut microbiota affects Dom and Sub social behavior. Sub mice inherit altered gut microbiota resulting in decreased SCFAs, overexpressed mucin secretion, and impaired immune T cell maturation with a reduction in colonic Tregs. This in turn lead to inflammation, shortens the colon, reduces mouse weight gain, and increases gut permeability. The increased gut permeability results in the infiltration of bacterial substances into the blood circuitry causing systemic inflammation, affecting the eWAT inflammatory state [28], possibly causing neuroinflammation, and altering the Sub mouse behavior.

Our data is the first to correlate early infancy gut microbiota composition, colon inflammation, and morphology abnormalities with social behavior deficits. This work reveals the significant role of the gut microbiota in early infancy in colon immunity, function, and social behavior. It may provide new insights for treating social behavior deficits through modulation of the gut barrier.

### Supplementary Information

**Additional file 1:** Colon Impairments and Inflammation Driven by an Altered Gut Microbiota Leads to Social Behavior Deficits Rescued by Hyaluronic Acid and Celecoxib.** Fig. S1.** Dominant submissive relationship (DSR) test of Dom and Sub mice. **Fig. S2.** Dom and Sub gut microbiome alpha diversity from early infancy to adulthood. **Fig. S3.** Gut microbiome of Dom and Sub mice from early infancy to adulthood. **Fig. S4.** Dom and Sub mouse liver and spleen measurements from early infancy. **Fig. S5.** Dom and Sub colon and spleen immune cells profile. **Fig. S6.** Colon cytokine arrays from Control and Hyaluronic Acid and Celecoxib- treated Sub mice. **Fig. S7.** Gut microbiome alpha and beta diversity upon HA and Celecoxib treatments of Sub mice. **Fig. S8.** Age-dependent colon tight junction gene expression in Dom and Sub mice.

## Data Availability

The datasets used and/or analyzed during the current study are available from the corresponding author on reasonable request.

## Abbreviations

AI: Anti-inflammatory
ASVs: Amplicon sequence variants
DOM: Dominant
DSR: Dominant–submissive relationship
EPM: Elevated plus maze
eWAT: Epididymis white adipose tissue
FISH: Fluorescence in situ hybridization
FITC: Fluorescein-5-isothiocyanate
FMT: Fecal microbiota transplantation
FST: Forced swim test
GF: Germ-free
H&E: Hematoxylin and eosin
HA: Hyaluronic acid
LDA: Linear discriminant analysis
LPS: Lipopolysaccharide
PAS: Periodic acid–Schiff
PCoA: Principal component analysis
SCFA: Short chain fatty acid
SD: Standard deviation
Sub: Submissive
TCST: Three-chamber sociability test
Treg: Regulatory T-cells
tTreg: Thymic Tregs
ZINB: Zero-inflated negative binomial
