# Supplementary material for: Colon impairments and inflammation driven by an altered gut microbiota leads to social behavior deficits rescued by hyaluronic acid and celecoxib
Source: BMC Med. 2024 Apr 29;22:182. doi: 10.1186/s12916-024-03323-0 (PMC11059729; doi:10.1186/s12916-024-03323-0)
Supplement: Supplementary file 1 — Additional file 1: Colon Impairments and Inflammation Driven by an Altered Gut Microbiota Leads to Social Behavior Deficits Rescued by Hyaluronic Acid and Celecoxib. Fig. S1. Dominant submissive relationship (DSR) test of Dom and Sub mice. Fig. S2. Dom and Sub gut microbiome alpha diversity from early infancy to adulthood. Fig. S3. Gut microbiome of Dom and Sub mice from early infancy to adulthood. Fig. S4. Dom and Sub mouse liver and spleen measurements from early infancy. Fig. S5. Dom and Sub colon and spleen immune cells profile. Fig. S6. Colon cytokine arrays from Control and Hyaluronic Acid and Celecoxib- treated Sub mice. Fig. S7. Gut microbiome alpha and beta diversity upon HA and Celecoxib treatments of Sub mice. Fig. S8. Age-dependent colon tight junction gene expression in Dom and Sub mice. [file 12916_2024_3323_MOESM1_ESM.docx]

# Colon Impairments and Inflammation Driven by an Altered Gut Microbiota Leads to Social Behavior Deficits Rescued by Hyaluronic Acid and Celecoxib

Agranyoni Oryan^1^, Sur Debpali^1^, Amidror Sivan^2^, Shidlovsky Nuphar^2^, Bagaev Anastasia^1^ Yissachar Nissan^2^, Pinhasov Albert^3*^, Navon-Venezia Shiri^3*^

^1^ Department of Molecular Biology, Ariel University, Ariel, Israel.

^2^ The Goodman Faculty of Life Sciences, Bar-Ilan Institute of Nanotechnology and Advanced Materials, Bar Ilan University, Ramat Gan, Israel.

^3^Department of Molecular Biology and the Dr. Miriam and Sheldon G. Adelson School of Medicine, Ariel University, Ariel, Israel.

*Equal contribution

**
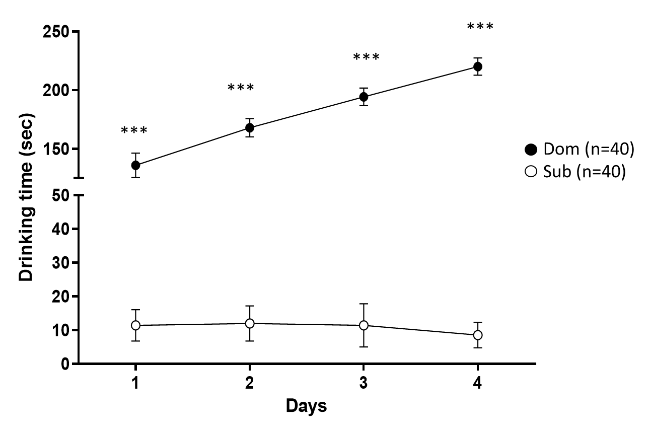

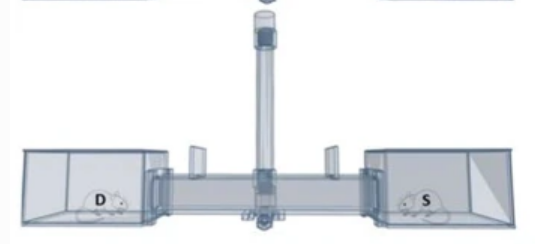
Supplementary Material**

B

A

Dom (n=40)

Sub (n=40)

**Fig. S1. Dominant submissive relationship (DSR) test of Dom and Sub mice.** (A) The DSR apparatus scheme adopted from Pinhasov et al. [1]. (B) Y-axis represents the drinking time measured during a 5 min period of testing of each mouse. Statistical significance was determined using a student's t-test, (***) p<0.001. Error bars present standard deviation.


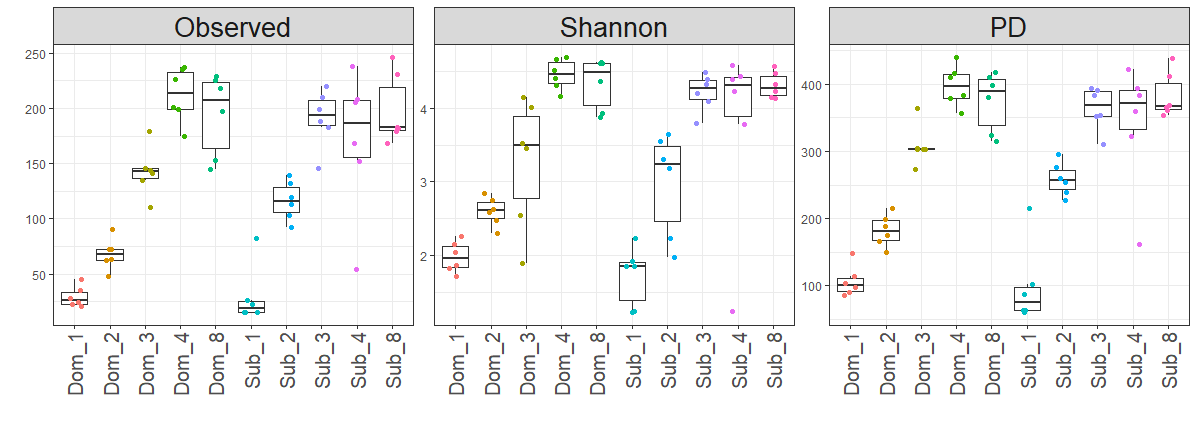


**Fig. S2. Dom and Sub gut microbiome alpha diversity** **from early infancy to adulthood**. Boxplots illustrating alpha diversity indices of Observed ASVs, Shannon-Weiner index, and PD, the Faith’s phylogenetic diversity, in bacterial gut microbiomes of 6 Dom and 6 Sub mice (3 males and 3 females) from 1 week old (designated Dom_1 or Sub_1) to 8 weeks (designated Dom_8 or Sub_8) old.

G

E

D

F

C

B

A

**Fig. S3. Gut microbiome of Dom and Sub mice from early infancy to adulthood.**

(A) A heatmap of the 100 most variant gut microbiome species identified in Dom mice at different ages; taxa with similar distributions are grouped. (B) A heatmap of the 100 most variant gut microbiome species identified in Sub mice at different ages; taxa with similar distributions are grouped. (C) Relative abundance of up to the genus level of Dom and Sub fecal samples at week 1, (D) week 2, (E) week 3, (F) week 4, and (G) week 8, showing changed (p<0.05) taxa between the groups. Statistical significance was assessed by using a student’s t-test.

B

A

**Fig. S4. Dom and Sub mouse liver and spleen measurements from early infancy**.

Liver and spleen tissues were removed from the same mice [Dom (n=53) and Sub (n= 51)] that were included in the eWAT and GI follow-up (see Fig. 2). (A) The average liver mass normalized to body weight follow-up. (B) The average spleen mass normalized to body weight follow-up. Error bars show standard deviations. m - males; f - female.


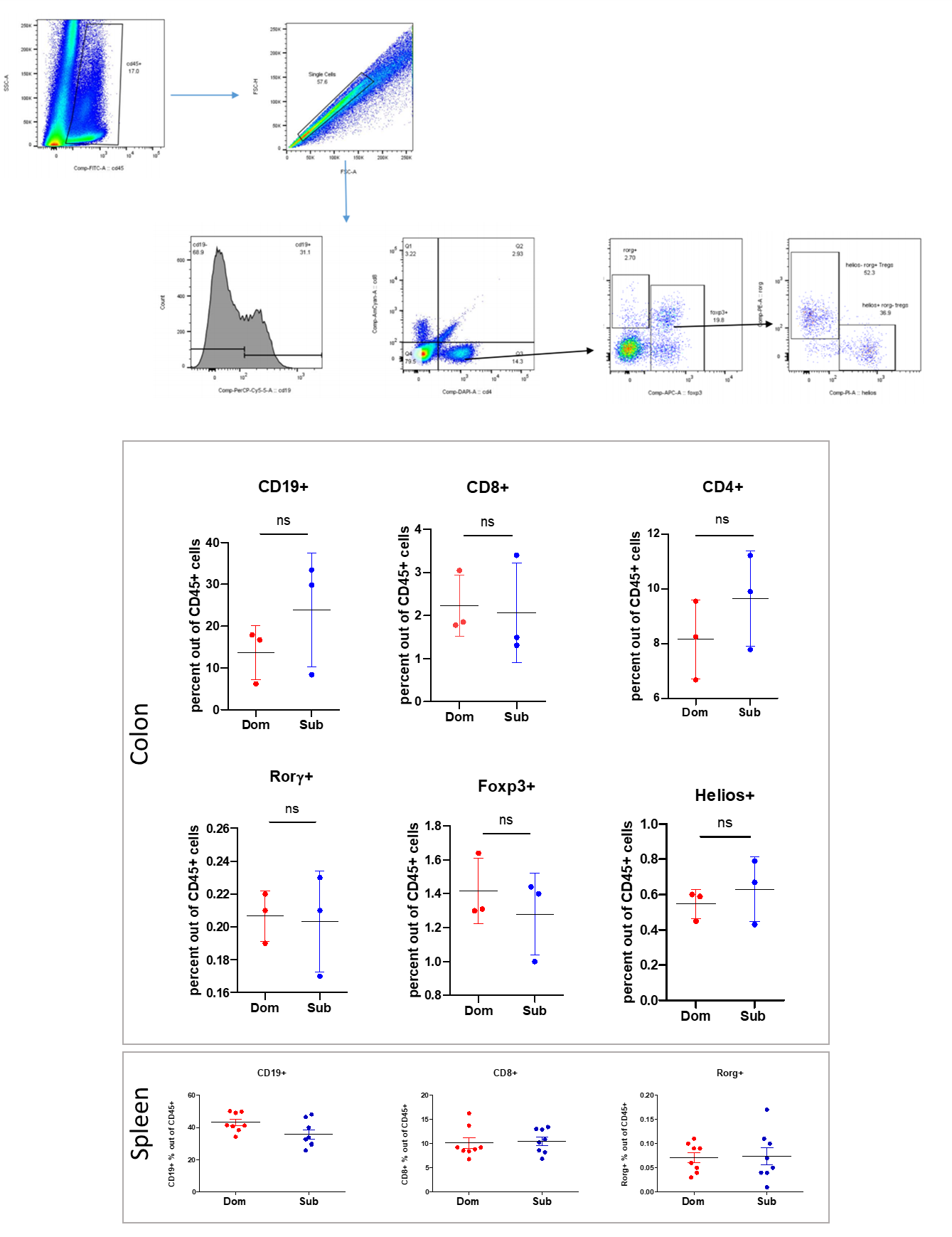


J

H

I

G

F

E

D

C

B

A

**Fig. S5. Dom and Sub colon and spleen immune cells profile.**

(A) The FACS method used to characterize the colon immune cells population. (B) Dom (n=3) and (B) Sub (n=3) mouse colons were stained with CD45+, CD19+, CD4+, CD8+, Rorg+, Foxp3+ and Helios+ fluorescent antibodies and were processed in a FACS machine. Percent of (B) CD19+, (C) CD8+, (D) CD4+ (E), Rorg+, (F) Foxp3+, (G) Foxp3+/Helios+. (H) Dom (n=8) and Sub (n=8) mouse spleens were labeled with CD45+, CD19+, CD4+, CD8+, Rorg+, Foxp3+ and Helios+ fluorescent antibodies and then were separated in a FACS machine. Percent of (H) CD19+, (I) CD8+, (J), Rorg+, out of CD45+ cells. Error bars represents standard deviations.


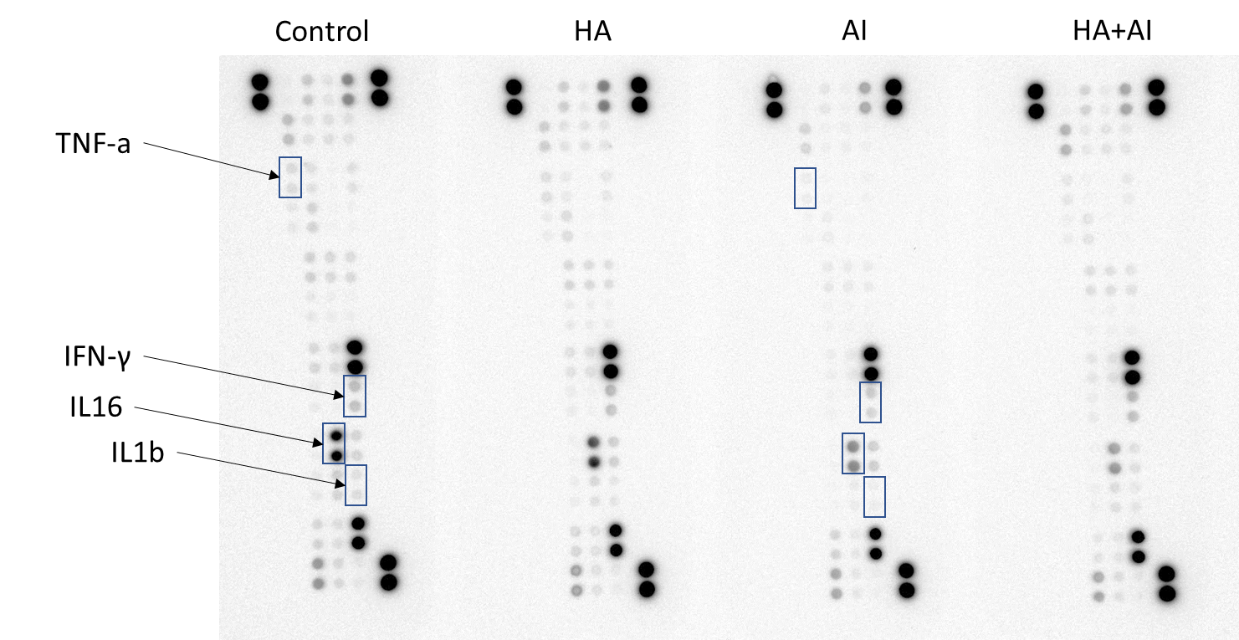


**Fig. S6. Colon cytokine arrays from Control and Hyaluronic Acid and Celecoxib- treated Sub mice.**

A cytokine array comparison of pooled proteins extracted from the colons of Sub control mice (n=3), HA-treated Sub mice (n=3), Celecoxib (AI)-treated Sub mice (n=3), and Sub mice treated with a combination of both agents (HA and AI) (n=3).


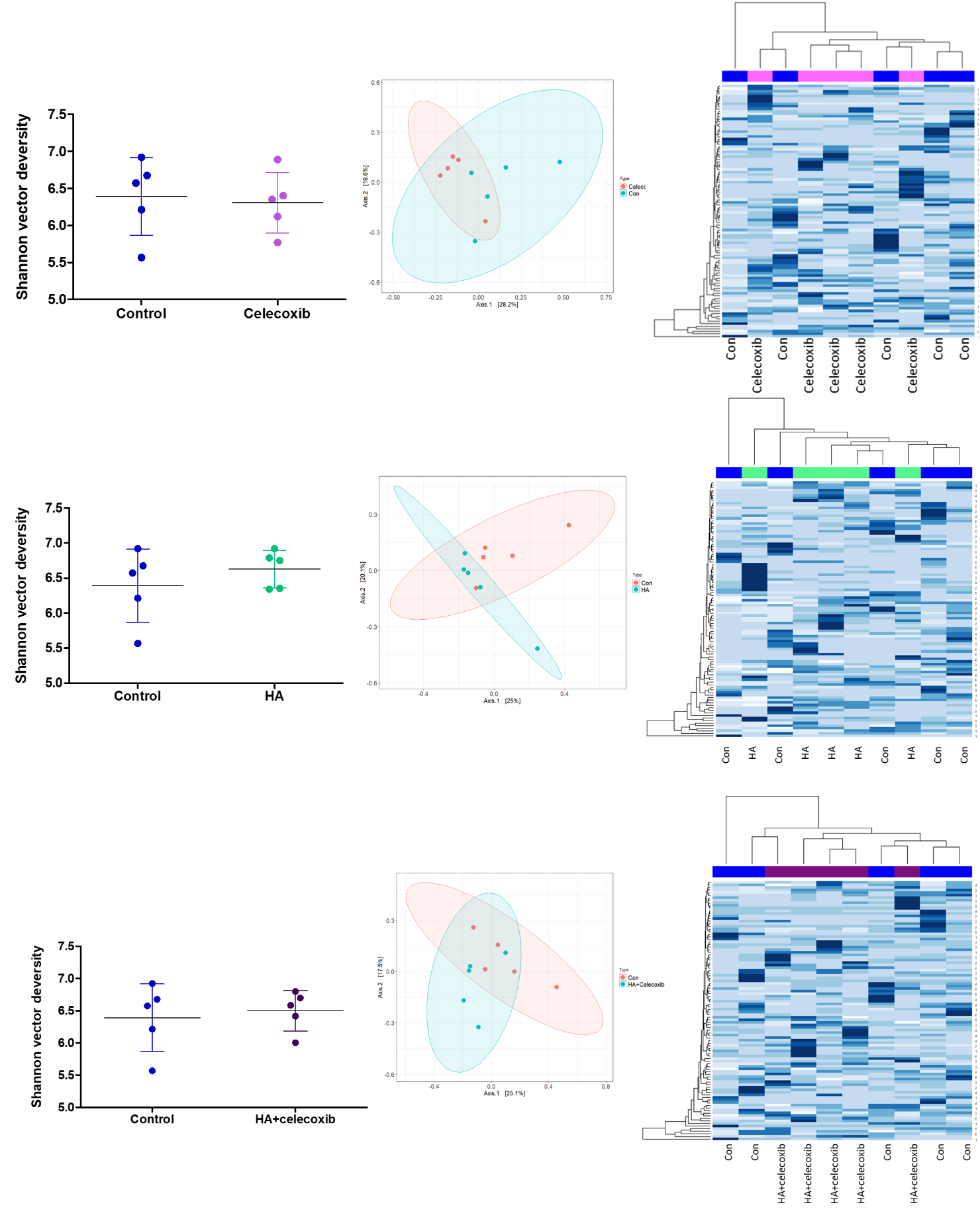


J

I

H

D

F

E

C

B

G

A

**Fig. S7. Gut microbiome alpha and beta diversity upon HA and Celecoxib treatments of Sub mice.** (A, D and G) - Alpha diversity of the gut microbiome of control and Celecoxib treated Sub mice; (B, E and H) - PCoA of control and HA treated Sub mice; (C, F and I) A heatmap of the 100 most variant species identified of Sub control and HA+ Celecoxib treated mice.

C

B

A

**Fig. S8. Age-dependent colon tight junction gene expression in Dom and Sub mice.** Expression of (A) Claudin-4 (CLD4) (B) Claudin-7 (CLD7), and (C) ZO3, normalized to HPRT in Dom (n=30 males) and Sub (n=34 males) mice, at the ages of 0, 1, 2, 3, 4, 8, and 12 weeks old. Statistical significance was determined using a student’s t-test, (*) p<0.05, (**) p<0.01, (***) p<0.001. Error bars show standard deviation.

**Reference**

1. Pinhasov A, Shmerkin E, Libergod L, Kirby M, Agranyoni O, Vinnikova L, et al. Development of a Selectively-Bred Mouse Model of Dominance and Submissiveness: Technical Considerations. In: Harro J, editor. Psychiatric Vulnerability, Mood, and Anxiety Disorders: Tests and Models in Mice and Rats. New York, NY: Springer US; 2023. p. 353–77.
